# Influence of ovarian torsion on reproductive outcomes and mode of delivery

**DOI:** 10.3389/fmed.2024.1370409

**Published:** 2024-03-27

**Authors:** Tali Silberstein, Amir Freud, Yael Baumfeld, Eyal Sheiner, Adi Yehuda Weintraub, Salvatore Andrea Mastrolia, Giuseppe Trojano, Eli Harris Bernstein, Polina Schwarzman

**Affiliations:** ^1^Department of Obstetrics and Gynecology, Soroka University Medical Center, Ben Gurion University of the Negev, Beer Sheva, Israel; ^2^Clinical Research Center, Soroka University Medical Center, Faculty of Health Sciences, Ben Gurion University of the Negev, Beer Sheva, Israel; ^3^Department of Obstetrics and Gynecology, Ospedale Madonna delle Grazie, Matera, Italy; ^4^The Medical School for International Health, Ben Gurion University of the Negev, Beer Sheva, Israel

**Keywords:** cesarean section, ovary, laparoscopy, laparotomy, pregnancy

## Abstract

**Purpose:**

To investigate differences in reproductive outcomes among patients before and following ovarian torsion.

**Study design:**

In this retrospective cohort study, we investigated the reproductive outcomes of patients who underwent surgery for ovarian torsion between 1988 and 2015 in a tertiary medical center. Data on deliveries before and after ovarian torsion were compared.

**Results:**

During the study period, 199 women underwent surgery due to ovarian torsion. The majority (91.4%; *n* = 182) underwent detorsion, and 8.6% (*n* = 17) underwent unilateral adnexectomy. At the time of the torsion, 27.6% (*n* = 55) of patients were pregnant. Among women who suffered from ovarian torsion, about half (52%) of the deliveries occurred before the torsion and 48% following the torsion. No significant difference in the live birth rate was noted (*p* = 0.19). The fertility treatment rate in our cohort was 7.5% before and 5% after the torsion (*p* = 0.01). In addition, live birth, cesarean delivery, and fertility treatment rates were similar in women who underwent detorsion vs. those who had adnexectomy.

**Conclusion:**

Surgically treated ovarian torsion does not appear to negatively influence fertility and live birth potential.

## Introduction

Adnexal torsion is defined as a rotation of the ovary and fallopian tube to varying degrees (1). The prevalence of adnexal torsion is about 2–6% (2, 3). Women are more prone to torsion in their reproductive years; however, it can occur in all age groups (1, 4–6).

The rotation of vascular pedicles may cause venous and lymphatic occlusion, which is followed by arterial insufficiency. The congestion may be followed by hemorrhagic infarction, gangrene, and, finally, necrosis (7). Ischemia–reperfusion injury, referring to the microvascular and parenchymal cell dysfunction of ischemic organs, can occur following the restoration of tissue perfusion subsequent to ischemia. This reperfusion injury is mediated by reactive oxygen species (ROS) generated via lipid peroxidation, promoting the release of inflammatory agents (7, 8).

Classically, ovarian torsion causes a dark blue-black discoloration of the ovary (7). In the past, oophorectomy has been the traditional treatment, likely due to the assumption that tissue that has lost its viability may cause damage through mediators of reperfusion or by embolism. This surgical approach was found to cause subfertility or early-onset menopause (9). In recent years, there has been a shift to a fertility sparing treatment, following studies that demonstrated viability of the ovary after detorsion (9–11).

Following this shift, questions rose regarding the possible negative impact of detorsion on ovarian reserve and viability of follicles.

In order to evaluate the impact of ovarian torsion on reproductive ability, in this study, we compared the reproductive outcomes in patients before and after surgery for ovarian torsion.

## Materials and methods

A retrospective cohort analysis was conducted at the Soroka University Medical Center between 1988 and 2015. We included all patients diagnosed with ovarian torsion by laparoscopy or laparotomy in our hospital during the study period.

Women who had torsion at age 17 or less or who by the year 2018 were not yet 20 years old were excluded.

We compared the reproductive data after the surgery (study cases) to reproductive data before the surgery (control cases) for the same study population.

Data were collected from the hospital computerized database. We reviewed all the patient history and operative reports, which are recorded by a Gynecologist immediately following the detorsion or adnexectomy procedure. Experienced medical secretaries routinely review the information prior to entering it into the database to ensure its maximal completeness and accuracy. Coding is performed after assessing medical records as well as routine hospital documents. We retrieved all patients with ICD 9 code of ovarian torsion and opened their files to retrieve information regarding general characteristics, surgical procedures, and obstetric outcomes. We defined fertility treatment according to ICD 9 diagnosis of *in vitro* fertilization, intrauterine insemination, or ovulation induction treatment.

Study outcomes were represented by live birth rate, mode of delivery after ovarian torsion, as well as the employment of assisted reproductive techniques. These were assessed before and after torsion, but also comparing women who underwent detorsion vs. those who had adnexectomy or those who underwent laparoscopy vs. those who underwent open surgery.

Statistical analysis was performed using the SPSS package 23 ed. (SPSS, Chicago, IL).

Categorical variables data are presented using a percentile, and statistical significance was tested using the X^2^ or Fisher’s exact test, as appropriate.

Numerical variables data are presented using median and interquartile range, and statistical significance was analyzed using the Mann–Whitney test.

Continuous variables data are presented using mean and standard deviation, and Student t-test was used for statistical analysis, paired *t*-test was used when appropriate. A *p*-value of <0.05 was considered statistically significant.

The Institutional Review Board of Soroka University Medical Center approved the study that has been performed in accordance with the ethical standards laid down in the 1964 Declaration of Helsinki and its later amendments.

The study was designed according to the STROBE (12) Statement checklist with items for cohort studies.

## Results

During the study period, we identified 199 women who underwent surgery due to ovarian torsion and met the inclusion criteria. The mean age of patients at the time of surgery was 25.95 ± 6.12 years. The demographic and general characteristics of the study population at the last delivery are presented in Table 1.

**Table 1:** Demographic and general characteristics of the study population at the last delivery.

Demographic characteristics	Value
Age at surgery	25.95 ± 6.12
Gravidity	3.69 ± 2.66
Parity	3.11 ± 2.37
Vaginal delivery	440 (82)
Cesarean delivery	98 (18)
Hypertension	2 (1)
Diabetes mellitus	9 (4.5)
Obesity	17 (8.5)
Thromboembolic disease	1 (0.5)
Prior surgery	2 (1)
Endocrine disease	6 (3)
Oncologic disease	4 (2)

Data is presented as mean ± standard deviation, number (percentage).

The vast majority (91.4%, *n* = 182) of the women underwent detorsion, with the remaining 8.6% (*n* = 17) underwent unilateral adnexectomy. The decision of performing adnexectomy depended on the presence of multiple torsion, ovarian aspect, and its recovery after torsion resolution and restoration of ovarian blood flow.

In 94.4% (*n* = 187) of the cases, the surgical approach was via laparoscopy, and the remaining 5.6% (*n* = 12) was via laparotomy (Table 2). Laparotomy was evaluated when large ovaries were detected due to the torsion, when the woman was pregnant or, even in absence of these conditions, if a skilled operator in laparoscopy was not available.

**Table 2:** Characteristics of surgical interventions in the study population.

Surgery characteristics	Value
Detorsion	182 (91.4)
Adnexectomy	17 (8.6)
Open Approach	12 (5.6)
Laparoscopy	187 (94.4)
Surgery during pregnancy	55 (27.6)

Data is presented as number (percentage).

At the time of the ovarian torsion, 27.6% (*n* = 55) of patients were pregnant.

Among all women who were operated for ovarian torsion, the 52% of deliveries (*n* = 280) occurred prior to the torsion (control cases) and 48% (*n* = 258) following the torsion (study group). No significant difference in the live birth rate was found between the study and control cases (*p* = 0.19; Table 3).

**Table 3:** Fertility and reproductive outcomes before and after ovarian torsion.

Variable	Pre-surgery cases	Post-surgery cases	*P* value
Live birth	280 (52)	258 (48)	0.19
Cesarean section	22 (8)	76 (29)	>0.001
Fertility treatments	15 (7.5)	10 (5)	0.01

Data is presented as number (percentage).

The time interval between surgical intervention and last delivery for the women in the study group was 4.84 ± 3.94 years.

The cesarean delivery (CD) rate was significantly higher in the study group compared to the control group [29% (*n* = 76) vs. 8% (*n* = 22), respectively, *p* < 0.001]. The evaluation of the indications for CD after ovarian torsion revealed that a significant part of them were relative indications for CD, meaning that, although they were eligible, they refused to undergo a vaginal delivery and requested to deliver by cesarean (Supplementary Table S1).

The fertility treatment rate in our cohort was 7.5% (*n* = 15) before and 5% (*n* = 10) after the torsion (*p* = 0.01; Table 3).

An additional analysis was performed to compare women who underwent adnexectomy versus those who underwent detorsion. No difference regarding fertility, CD after the procedure, and deliveries after the procedure was found for the study groups (Table 4). No cases of recurrence of ovarian torsion were described in women undergoing detorsion.

**Table 4:** Comparison between women who underwent adnexectomy versus those who underwent detorsion.

Variable	Adnexectomy	Detorsion	*P* value
Live birth after surgery	9 (59)	129 (77.8)	0.343
Cesarean section after surgery	6 (33)	53 (29)	0.709
Fertility treatments	1 (5.6)	8 (4.4)	0.821

Data is presented as number (percentage).

Then, we compared laparoscopy to open procedure. No difference regarding fertility and deliveries after surgery was found for the study groups. Interestingly, we noted that open surgery group had a significantly higher CD rate than the laparoscopic group [64% (*n* = 7) vs. 28% (*n* = 52), *p* value = 0.012] (Table 5).

**Table 5:** Comparison between women who underwent laparoscopy versus those who underwent open surgery.

Variable	Laparoscopy	Open surgery	*P* value
Live birth after surgery	5 (45)	122 (65)	0.184
Cesarean section after surgery	52 (28)	7 (64)	0.012
Fertility treatments	8 (4.3)	1 (9.1)	0.456

Data is presented as number (percentage).

## Discussion

A retrospective cohort analysis was performed in order to determine whether ovarian torsion affects the mode of delivery, live birth rate, and the employment of assisted reproductive techniques. Adnexal torsion is a common gynecological emergency. The ovary carries the entire follicle cohort from embryonal life, therefore holding the entire fertility potential (13, 14). In recent years, the surgical treatment of ovarian torsion has been more conservative (15).

Ischemia–reperfusion injury is defined as the paradoxical worsening of cellular dysfunction and death following restoration of blood flow to previously ischemic tissues. The re-establishment of blood flow is essential to the salvage of ischemic tissues. However, reperfusion itself paradoxically causes further damage, threatening function and viability of the organ (16). Activated endothelial cells in all segments of the microcirculation produce more oxygen radicals but less nitric oxide in the initial period following reperfusion. The resulting imbalance between superoxide and nitric oxide in endothelial cells leads to the production and release of inflammatory mediators (e.g., platelet-activating factor, tumor necrosis factor) and enhances the biosynthesis of adhesion molecules that mediate leukocyte-endothelial cell adhesion (17). Thus, early diagnosis of ovarian torsion is crucial, as unrelieved torsion may progress to hemorrhagic infarction, peritonitis, and infertility due to ischemia of adnexal structures (18).

It has become clear that the appearance of the ovary during surgery does not correlate well with subsequent ovarian recovery and function (10, 11, 19). According to current guidelines, oophorectomy is unnecessary in cases of black discoloration of the ovary seen during surgery (20).

In our study group, we found no negative impact of ovarian torsion on the live birth rate.

A decrease in the prevalence of fertility treatments between study and control cases was demonstrated (15% before vs. 10% after the torsion; *p* = 0.01). This data is interesting if we consider the fact that, when trying to conceive after the ovarian torsion, women were older. Reduction in fertility treatment cases after surgery in an older patient is a positive finding but may be explained by possible bias. It might be that some of the women who were sub-fertile had finished their reproductive plan and therefore did not need further fertility treatments. In addition, it is likely that, following a complication such as torsion, physicians and patients may refrain from ovulation induction, which is a risk factor for torsion (11). Another possible explanation could derive from the health system policy in Israel of funding fertility treatments only for the first two born children.

Ovarian cysts are a known risk factor for ovarian torsion, especially if larger than five centimeters (21). There are still debates between surgeons and fertility specialists regarding the safety of cystectomy for ovarian reserve. Today, it is customary in ovarian torsion with a prominent cyst to perform ovarian cystectomy (22). In a study on patients who underwent detorsion and ovarian cystectomy during the same procedure, no negative impact on the pregnancy rate or the live birth rate was found compared to controls (23). In our population, cystectomy was performed in only few cases, therefore we did not enter this parameter for analysis.

Something needs to be said about the clinical implications of our findings. Ovarian torsion is not an indication for cesarean delivery after detorsion or adnexectomy. Although there was an increase in the cesarean delivery rate after ovarian torsion, this is a data that deserves a second thought but should not be considered as a result of torsion itself. Indeed, this might be a consequence of women’s request for not undergoing a vaginal delivery due to the fear for previous surgical procedure, especially if performed during pregnancy or in those women whose pregnancy was achieved after fertility treatments. Moreover, the limited sample size could also be responsible for this observation.

Lastly, fertility was not affected by ovarian torsion, although anti-Mullerian hormone level and antral follicle count would be more reliable markers of ovarian reserve and, consequently, of reproductive outcome.

Due to all the above ovarian torsion is a clinical condition that should be treated as it is without providing specific recommendations regarding the management of the ongoing or future pregnancies.

### Strength and limitations of the study

Our Institution is the only hospital in the Negev (Southern Israel) thus, the study is based on non-selective population data. The fact that our hospital is the only tertiary hospital in the entire region enabled us to combine the gynecological and obstetrical databases with minimal missing data.

There are several limitations to our study that have to be acknowledged. We evaluated the reproductive outcomes and not the ovarian function directly, and thus we cannot exclude the possibility that the women ovulated more from the “healthy” ovary, thereby masking possible damage to the affected ovary. In addition, the lack of data related to male factor infertility or mechanical factor, as well as the existence of confounding factors such as age, may be important power limiting factors. Furthermore, the study population size is relatively small, and the retrospective nature of the study has its inherent limitations. For example, the study includes data from a broad time interval (1988–2015) in which both surgical approaches and assisted reproductive fertility treatments underwent changes.

Future prospective and larger studies are needed in order to confirm our results.

In conclusion, surgically treated ovarian torsion does not seem to impair fertility or live birth potential.

## Data availability statement

The raw data supporting the conclusions of this article will be made available by the authors, without undue reservation.

## Ethics statement

The studies involving humans were approved by Soroka University Medical Center IRB. The studies were conducted in accordance with the local legislation and institutional requirements. Written informed consent for participation was not required from the participants or the participants’ legal guardians/next of kin in accordance with the national legislation and institutional requirements.

## Author contributions

TS: Conceptualization, Writing – review & editing. AF: Investigation, Writing – original draft. YB: Data curation, Writing – review & editing. ES: Methodology, Writing – review & editing. AW: Supervision, Writing – original draft. SM: Writing – original draft. GT: Supervision, Writing – review & editing. EB: Writing – original draft. PS: Supervision, Writing – review & editing.
