# Supplementary material for: Influence of ovarian torsion on reproductive outcomes and mode of delivery
Source: Front Med (Lausanne). 2024 Mar 27;11:1370409. doi: 10.3389/fmed.2024.1370409 (PMC11005820; doi:10.3389/fmed.2024.1370409)
Supplement: Supplementary file 1 [file Table_1.DOCX]

**Supplementary Table 1.** Indications to cesarean delivery after surgery for ovarian torsion.

| **Indication to cesarean delivery** | **n=76** |
| --- | --- |
| Placenta previa | 1 (1.3) |
| Previous multiple cesarean delivery | 23 (3.03) |
| Non reassuring fetal heart rate | 3 (3.9) |
| Breech presentation | 5 (6.6) |
| Not available data | 9 (11.8) |
| Multiple pregnancy | 4 (5.3) |
| Suspected macrosomia | 5 (6.6) |
| Non progressive labor | 7 (9.2) |
| Previous single cesarean delivery | 9 (11.8) |
| Pregnancy after in vitro fertilization | 10 (13.1) |

*Data is presented as number (percentage)*
